# Temporal Evolution of Optic Nerve Sheath Diameter/Eyeball Ratio on CT and MRI for Neurological Prognostication After Cardiac Arrest

**DOI:** 10.3390/jcm14196891

**Published:** 2025-09-29

**Authors:** Jiyoung Choi, So-Young Jeon, Jung Soo Park, Jin A Lim, Byung Kook Lee

**Affiliations:** 1 Department of Emergency Medicine, College of Medicine, Chungnam National University, Daejeon 35015, Republic of Korea; clfgus311@hanmail.net (J.C.); chloe9899@naver.com (S.-Y.J.); 2 Department of Emergency Medicine, Chungnam National University Hospital, Daejeon 35015, Republic of Korea; 3 Department of Biomedical Laboratory Science, The Graduate School, Yonsei University, Seoul 03722, Republic of Korea; jin-a0070@hanmail.net; 4 Department of Emergency Medicine, Chonnam National University Medical School, Chonnam National University Hospital, Gwangju 61469, Republic of Korea; bbukkuk@hanmail.net

**Keywords:** out-of-hospital cardiac arrest, prognosis, optic nerve sheath diameter, computed tomography, magnetic resonance imaging, neuroprognostication

## Abstract

**Background:** Optic nerve sheath diameter (ONSD) and its ratio to eyeball transverse diameter (ETD; ONSD/ETD) are potential markers for elevated intracranial pressure in comatose survivors of out-of-hospital cardiac arrest (OHCA). However, their prognostic accuracy remains uncertain. We compared their predictive value via compted tomography (CT)and magnetic resonance imaging (MRI) before and after targeted temperature management (TTM) in OHCA survivors. **Methods:** This retrospective study included adult comatose OHCA survivors who underwent TTM and serial brain imaging. ONSD and ONSD/ETD ratios were measured on brain CT and MRI at two predefined time-points: within 6 h (pre-TTM) and at 72–96 h (post-TTM) after return of spontaneous circulation. Intra-rater reliability was assessed using intraclass correlation coefficients (ICC). Poor neurological outcome was defined as a Cerebral Performance Category score of 3–5 at 6 months. Prognostic performance was evaluated using area under the receiver operating characteristic curve (AUC). **Results:** Among 136 patients, 78 (57%) had poor neurological outcomes. Only ONSD (5.12 vs. 5.37 mm) and ONSD/ETD ratio (0.22 vs. 0.23) measured on post-TTM MRI were significantly higher in the poor outcome group. These results depicted modest predictive performance (AUC, 0.67 and 0.65, respectively), whereas all CT-based and early MRI measurements had AUC < 0.60. Intra-rater reliability for ONSD and ETD was higher on CT (ICC: up to 0.93) than on MRI (ICC: 0.73–0.80). **Conclusions:** Delayed MRI-based ONSD and ONSD/ETD showed statistically significant but modest prognostic value, with limited clinical applicability as a stand-alone tool. These findings underscore the relevance of measurement timing, supporting ONSD as an adjunctive, rather than definitive, tool in multimodal prognostication.

## 1. Introduction

The number of patients treated for cardiac arrest in Korean emergency facilities increased steadily from 2018 to 2022 [1]. Out-of-hospital cardiac arrest (OHCA) carries high mortality rates and significant neurological sequelae [2,3,4].

Despite advancements in prehospital emergency care systems, survival rates for OHCA remain low [5,6]. Furthermore, a significant proportion of survivors experience severe neurological impairment, particularly those with hypoxic–ischemic brain injury (HIBI) accompanying OHCA [7].

In survivors of cardiac arrest, brain edema resulting from HIBI significantly contributes to elevated intracranial pressure (ICP), a critical factor associated with poor neurological outcomes [8,9,10]. Although direct ICP measurement remains the gold standard, its invasive nature and associated risks make it less desirable in many clinical scenarios [11,12,13].

Optic nerve sheath diameter (ONSD) enlargement is thought to reflect elevated ICP owing to the anatomical continuity of the subarachnoid space extending along the optic nerve sheath. Recent studies have explored the utility of ONSD measurement via ultrasound, computed tomography (CT), and magnetic resonance imaging (MRI) as a predictive tool for neurological outcomes following cardiac arrest [11,12,14,15,16]. However, findings have varied, indicating the need for further investigation. In particular, it remains unclear whether adjusting ONSD by eyeball size (i.e., ONSD/ETD ratio) provides additional prognostic value. Moreover, although CT and MRI are used in clinical practice, direct comparisons of their performance in ONSD-based prognostication remain limited. Whether delayed ONSD measurements better reflect secondary brain edema and correlate more strongly with neurological outcomes also warrants further investigation. Additionally, there is little evidence on the association between ONSD-based metrics and serum biomarkers such as neuron-specific enolase (NSE), a validated marker of HIBI.

This study aimed to compare the predictive accuracy of the ONSD and its ratio to the eyeball transverse diameter (ONSD/ETD) when measured using CT and MRI before and after TTM in OHCA survivors. We additionally analyzed whether ONSD and ONSD/ETD ratios correlate with serum NSE levels measured at corresponding time points, as an indicator of HIBI severity. Finally, to verify consistency between the modalities, a single rater measured ONSD/ETD on CT and MRI twice to assess intra-rater reliability.

## 2. Materials and Methods

### 2.1. Study Design and Population

In this retrospective cohort study, we analyzed prospectively collected registry data from adult comatose survivors of OHCA who underwent TTM at Chungnam National University Hospital (CNUH) in Daejeon, Republic of Korea, between May 2018 and December 2023. A subset of this data, encompassing 120 patients treated between May 2019 and December 2022, partially overlaps with data used in a previously published study exploring the relationship between ultra-early diffusion-weighted imaging and neurological outcomes [17]. The Institutional Review Board of CNUH reviewed and approved this retrospective cohort study (Approval No.: CNUH-2023-03-089; approved on 12 June 2023).

Inclusion criteria encompassed adult patients (≥18 years) who survived OHCA, underwent TTM, and had MRI scans performed either prior to or following TTM. The exclusion criteria were: patients who experienced cardiac arrest caused by trauma, those with significant artifacts or evidence of severe brain atrophy or sequelae from prior injuries (such as stroke, tumor, or intracerebral hemorrhage) on MRI scans, those whose first MRI scan was conducted more than 6 h after return of spontaneous circulation (ROSC), those whose second MRI scans were performed outside the 72–96 h post-ROSC window, and those who received extracorporeal membrane oxygenation (ECMO).

### 2.2. TTM Protocol

All patients included in this study underwent TTM. For 24 h, their temperature was maintained at either 33 or 36 °C using a feedback-controlled surface cooling system (Arctic Sun^®^ 5000; BD, Franklin Lakes, NJ, USA). Prior to March 2022, the institutional protocol uniformly set the target temperature at 33 °C; after that date, the attending physician determined the target temperature, selecting either 33 or 36 °C based on the patient’s hemodynamic stability and cardiac arrest etiology (cardiac vs. non-cardiac origin or shockable vs. non-shockable). This range aligns with the contemporary view that optimal benefit depends on early initiation, adequate depth (≈32–34 °C), and fever prevention up to 37.5 °C [18]. Following the maintenance phase of TTM, patients were gradually rewarmed to 37 °C at a controlled rate of 0.25 °C/h. Throughout the TTM process, patients received sedative agents and neuromuscular blockade. Additionally, they were provided standard intensive care based on international guidelines for post-cardiac arrest management, adapted to the institutional protocol. However, some patients were pronounced deceased based on circulatory or neurological criteria despite maximal support. In South Korea, withdrawal of life-sustaining therapy (WLST) was legally prohibited prior to February 2018 unless brain death was confirmed. Even after legalization, WLST remains uncommon in clinical practice owing to prevailing cultural, ethical, and societal norms [19,20]. At our institution, WLST is generally not performed during TTM unless brain death is confirmed and organ donation is being considered.

### 2.3. Data Collection

The data extracted from a prospective registry included age, sex, Charlson Comorbidity Index (CCI), witnessed arrest status (witnessed vs. unwitnessed), bystander-performed cardiopulmonary resuscitation (CPR) (yes vs. no), initial monitored rhythm (shockable vs. non-shockable), cardiac arrest etiology (cardiac vs. non-cardiac), no-flow time (interval from collapse to CPR), low-flow time (interval from CPR to ROSC), time to MRI and/or CT imaging (the time from ROSC to image acquisition), revised Cardiac Arrest Syndrome for Therapeutic hypothermia (rCAST), serum NSE levels obtained within 6 h and 72–96 h post-ROSC and the gray-white matter ratio (GWR) on CT scans. Missing data were not imputed; patients with unavailable measurements at a given timepoint were excluded from the respective receiver operating characteristic (ROC) analyses.

### 2.4. Measurement of ONSD and ETD in Brain CT and MRI

**Figure 1:**
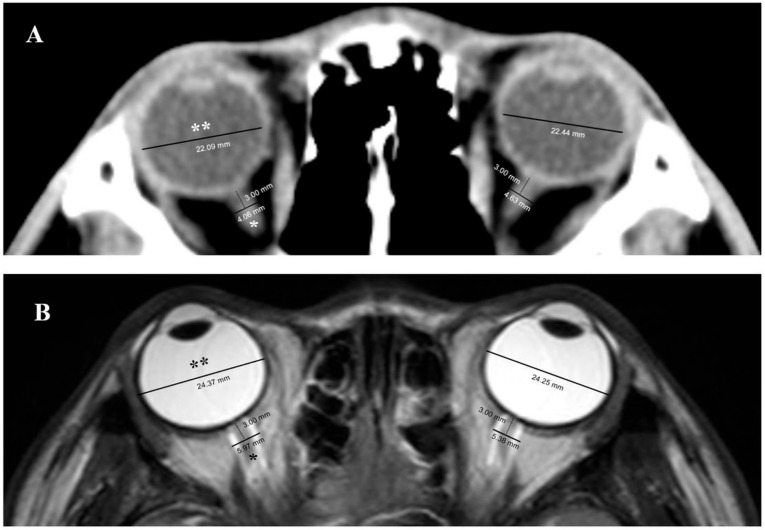
Measurement of the optic nerve sheath diameter and eyeball transverse diameter using computed tomography (**A**) and magnetic resonance imaging (**B**). The optic nerve sheath diameter (*) was measured using electronic calipers positioned 3 mm posterior to the globe along a perpendicular vector, while the eyeball transverse diameter (**) was determined by measuring from the retina to retina.

Our TTM protocol recommends, but does not mandate, performing two brain CT and MRI scans: one within 6 h (first) and another 72–96 h (second) after ROSC. MRI scans were performed using a 3T scanner (Intera Achieva; Philips Healthcare, Best, The Netherlands), including diffusion-weighted imaging (DWI), apparent diffusion coefficient (ADC) maps, T2-weighted imaging, and an axial proton density/T2-weighted turbo spin-echo fat-suppressed sequence. The scanning parameters were as follows: repetition time, 3000 ms; echo time, 80 ms; slice thickness, 3 mm; and spacing between slices, 4 mm. On the axial T2-weighted turbo spin-echo fat-suppressed images, the optic nerve sheath appeared as a high-signal area surrounding a low-signal region corresponding to the optic nerve. The ONSD was calculated using electronic calipers positioned 3 mm behind the globe in a perpendicular vector, whereas the ETD was measured from retina to retina. Similarly, CT scans were obtained in 5-mm slices using a 64-channel system (Somatom Sensation 64, Siemens Healthineers, Munich, Germany). The ONSD and ETD were measured as the distances between the outer margins of the thick sheath layers surrounding the optic nerve and globe, respectively. For ONSD, measurements were taken 3 mm behind the globe on both sides. Bilateral measurements for ONSD and ETD were performed for MRI and CT (Figure 1).

All measurements were performed by a single operator, who was thoroughly trained in the measurement technique and remained blinded to the patients’ clinical status. The mean values for ONSD and ETD were calculated by averaging the measurements obtained from both eyes, followed by the calculation of the ONSD/ETD ratio. All measurements were repeated at different time-points by the same investigator (J.C.).

### 2.5. NSE Measurement

Our institution’s TTM protocol includes the assessment of serum NSE levels at multiple time-points following ROSC: immediately after (H0) and at 72 h (H72). All samples were processed and analyzed at the same facility (GC Labs, Yongin-si, Republic of Korea). Any aliquots that were heavily contaminated with blood or contained hemolyzed blood were excluded from analysis. NSE concentrations were quantified using an electrochemiluminescence immunoassay kit (COBAS e801, Roche Diagnostics, Basel, Switzerland). The measurable concentration range for NSE was 0.1–300 ng/mL.

### 2.6. Outcomes

Neurological outcomes were assessed 6 months after the ROSC using the Glasgow–Pittsburgh Cerebral Performance Category (CPC) scale and were dichotomized as good (CPC 1–2) or poor (CPC 3–5). Outcomes were determined through face-to-face or telephone interviews conducted by an emergency physician or a neurologist who was fully informed of the study protocol and blinded to the patient’s prognosis. A poor neurological outcome was designated as the primary endpoint.

### 2.7. Statistical Analysis

Categorical variables are presented as counts with percentages, and continuous variables as means with standard deviations or medians with interquartile ranges, depending on normality assessed using the Shapiro–Wilk test. Comparisons of categorical variables were performed using χ^2^ tests with continuity correction for 2 × 2 tables or Fisher’s exact test, as appropriate. Continuous variables were compared using the Student’s *t*-test or Mann–Whitney U-test, depending on data distribution. Paired analyses of the first and second ONSD and ONSD/ETD measurements were conducted using the paired *t*-test or Wilcoxon signed-rank test. ROC curves were constructed to evaluate the predictive performance for poor outcomes at 6 months, with optimal cut-off values identified using Youden’s index. Prognostic performance was classified based on AUC values as follows: fail (0.50–0.59), poor (0.60–0.69), fair (0.70–0.79), considerable (0.80–0.89), and excellent (0.90–1.00) [21]. The comparative performance of predictors was assessed using the DeLong test [22]. Intra-rater reliability for continuous variables, including ONSD and ONSD/ETD ratios, was assessed using the intraclass correlation coefficient (ICC). ICC values were interpreted as follows: <0.50, poor; 0.50–0.75, moderate; 0.75–0.90, good; and >0.90, excellent reliability [23]. All statistical analyses were conducted using SPSS version 24 (IBM Corp., Armonk, NY, USA) and MedCalc^®^ Statistical Software version 20.118 (MedCalc Software Ltd., Ostend, Belgium). Statistical significance was defined as *p*-values < 0.05.

## 3. Results

### 3.1. Patient Characteristics

**Figure 2:**
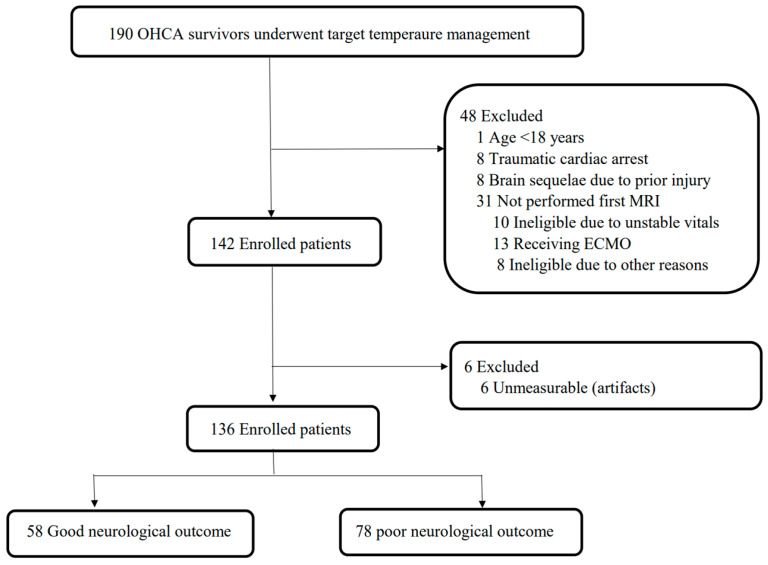
Flow diagram of the patients included in this study. OHCA, out-of-hospital cardiac arrest; ECMO, extracorporeal membrane oxygenation; MRI, magnetic resonance imaging.

Among 190 comatose OHCA survivors treated with TTM during the study period, 136 patients were included in our study after excluding non-adult patients (*n* = 1), those with traumatic cardiac arrest (*n* = 8), those with pre-existing brain injury leading to sequelae prior to cardiac arrest (*n* = 8), those who did not undergo the first MRI examination (*n* = 31), and those in whom ONSD or ONSD/ETD ratio measurements were not feasible owing to artifacts or anatomical abnormalities (*n* = 6). At 6 months post-ROSC, 58 patients (43%) had good neurological outcomes, while 78 (57%) had poor neurological outcomes (Figure 2). The demographic and clinical characteristics of patients, stratified according to neurological outcome at 6 months, are summarized in Table 1. Compared to the good neurological outcome group, the poor neurological outcome group demonstrated lower rates of witnessed events, bystander CPR, shockable rhythm, and cardiac etiology. Additionally, the poor neurological outcome group exhibited longer no- and low-flow times, lower GWR values, a higher proportion of females, and higher rCAST and serum NSE levels at H(0) and H(72). However, there were no significant differences between the two groups regarding the time interval from ROSC to the first and second CT and MRI scans, nor in age or CCI (Table 1).

### 3.2. Measurement of ONSD and ETD in Brain CT and MRI

The ICC values for ONSD measurements on CT exhibited good-to-excellent reliability during both the first (ICC = 0.93 [left], 0.92 [right]) and the second (ICC = 0.79 [left], 0.86 [right]) CT scans. Additionally, the ETD measurements demonstrated good reliability during the first CT scan (ICC = 0.75 [left], 0.76 [right]) and excellent agreement during the second CT scan (ICC = 0.82 [left], 0.87 [right]).

For MRI, the ICC values for ONSD measurements indicated good reliability during the first MRI scan (ICC = 0.80 [left], 0.78 [right]). However, during the second MRI scan, good reliability was observed for the left side (ICC = 0.77), whereas the right side demonstrated moderate agreement (ICC = 0.73). Additionally, the ICC values for ETD measurements showed moderate reliability for both sides during the first MRI scan (ICC = 0.73 [left], 0.74 [right]). In contrast, the second MRI scan indicated good reliability for both sides (ICC = 0.77 [left], 0.78 [right]) (Table 2).

### 3.3. ONSD and ONSD/ETD Ratios and Neurological Outcomes

Table 3 and Table 4 demonstrate that only the second MRI-measured ONSD (5.12 vs. 5.37, *p* = 0.003) and ONSD/ETD ratio (0.22 vs. 0.23, *p* = 0.01) were significantly higher in the poor neurological outcome group compared to the good neurological outcome group. Conversely, the ONSD and ONSD/ETD ratio measured from the first CT and MRI, as well as second CT, exhibited no significant differences between the two groups. Furthermore, in the good neurological outcome group, significant reductions were observed between the first and second MRI in both ONSD (5.31 vs. 5.12, *p* < 0.001) and ONSD/ETD ratio (0.23 vs. 0.22, *p* = 0.005). Contrastingly, no significant changes were found in the poor neurological outcome group.

### 3.4. Prognostic Performance of ONSD and ONSD/ETD Ratio on First and Second CT and MRI

Table 5 demonstrates the prognostic performance of ONSD and ONSD/ETD ratio measured from the first and second CT and MRI scans for predicting neurological outcomes at 6 months after ROSC. According to ROC analysis, ONSD and ONSD/ETD measured from the first and second CT, and the first MRI, showed poor prognostic performance (all AUC < 0.60). However, ONSD and ONSD/ETD ratio measured from the second MRI demonstrated prognostic performance ranging from fail to fair (AUC = 0.67; 95% confidence interval [CI], 0.56–0.78, and AUC = 0.65; 95% CI, 0.55–0.75).

### 3.5. Correlation Between NSE and ONSD and ONSD/ETD Ratios

**Figure 3:**
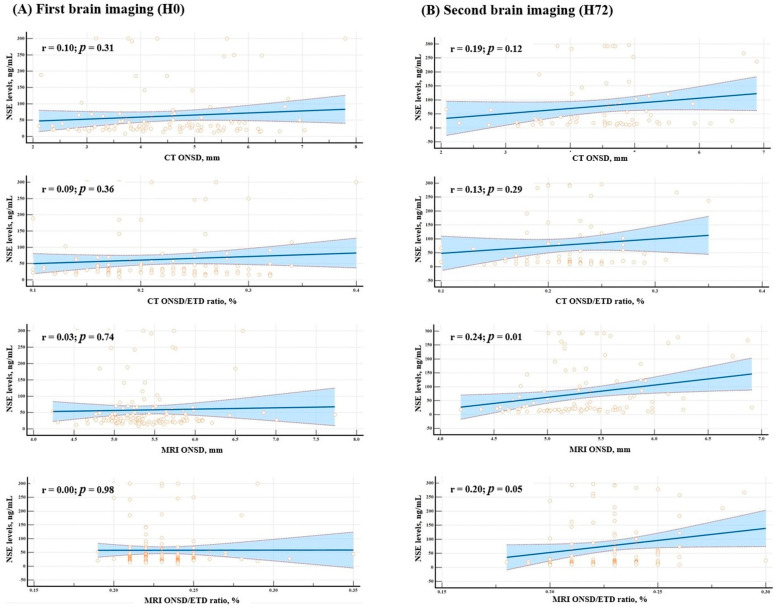
Correlations between serum neuron-specific enolase (NSE) levels and optic nerve sheath diameter (ONSD) or ONSD/eyeball transverse diameter (ETD) ratios measured on brain CT and MRI at H0 and H72. (**A**) At H0, no significant correlations were observed between NSE and ONSD or ONSD/ETD on CT or MRI. (**B**) At H72, a weak correlation was observed between NSE and MRI-derived ONSD (r = 0.24; *p* = 0.01), whereas correlations with ONSD/ETD and CT-derived measures were non-significant. Orange circles indicate individual patient data points. The blue line represents the regression line, and the shaded area denotes the 95% confidence interval. Units—NSE: ng/mL; ONSD: mm; ONSD/ETD: unitless.

We analyzed the correlations between NSE levels measured at H0 and H72 and ONSD and ONSD/ETD ratios obtained from CT and MRI at corresponding time-points. At H0, NSE showed no significant correlation with either ONSD or ONSD/ETD ratios measured by CT or MRI (*p* = 0.31, *p* = 0.36, *p* = 0.74, and *p* = 0.98, respectively; Figure 3A). At H72, NSE exhibited a fair positive correlation with ONSD measured by MRI (r = 0.24, *p* = 0.01; Figure 3B), while no correlation was observed with ONSD or ONSD/ETD ratios measured using CT, or with ONSD/ETD ratios measured by MRI (*p* = 0.12, *p* = 0.29, and *p* = 0.05, respectively; Figure 3B).

## 4. Discussion

In this cohort, only delayed MRI-based ONSD and ONSD/ETD ratios were associated with poor neurological outcomes, albeit with modest accuracy, whereas CT- and early MRI-derived measures showed no prognostic value. A weak correlation with H72 NSE further supports ONSD as a supplementary marker within a multimodal strategy, most informative when interpreted alongside biomarkers, DWI/ADC, and GWR. Clinically, ONSD should not be used to guide WLST decisions in isolation, and its value appears greatest when measured on delayed MRI (72–96 h post-ROSC), rather than during the ultra-early phase.

Kim et al. suggested a strong association between early CT-based ONSD (within 1 h post-ROSC) and poor neurological outcome (AUC = 0.93), showing high specificity and sensitivity when combined with GWR [15]. Conversely, studies by Lee et al. and Rush et al. found no significant relationship between early CT-derived ONSD and neurological outcome [24,25]. Our findings are consistent with studies that reported no significant association between early ONSD measurements and neurological outcomes, suggesting that early imaging may be insufficient to detect clinically relevant intracranial changes.

To address the limitations of raw ONSD measurements and account for interindividual anatomical variability, the ONSD/ETD ratio has been proposed as a normalized metric. Cho et al. recently evaluated this ratio in OHCA survivors and found it to have a slightly higher predictive value than ONSD alone (AUC = 0.66) [26]. Nonetheless, this performance remained limited, and the ratio was not independently associated with outcome in multivariable analyses. Similarly, herein, the ONSD/ETD ratio on delayed MRI showed modest predictive performance (AUC = 0.65), suggesting that normalization may improve measurement consistency without substantially enhancing prognostic accuracy. Notably, this association was absent in early imaging and CT.

Our findings emphasize the critical role of timing in the utility of ONSD-based prognostication. Cerebral edema and raised ICP may not reach their peak immediately post-ROSC, but instead continue to progress during the rewarming phase of TTM and beyond [11,12,13]. Park et al. reported that ONSD measured via ultrasound at 24 h showed better prognostic discrimination than measurements taken immediately post-ROSC [11]. Similarly, Song et al. observed a delayed rise in cerebrospinal fluid pressure in patients with poor neurological outcomes, supporting the time-dependent nature of intracranial changes following arrest [13]. Our study confirms this trajectory: ONSD values diverged between outcome groups only on MRI performed at 72–96 h, and not during the ultra-early phase. This suggests that a sufficient interval must elapse for ONSD to reflect clinically meaningful differences in intracranial pathology. The timing of MRI acquisition is clinically important. Current international guidelines recommend that neuroprognostication should not be performed earlier than 72 h after ROSC, and decisions such as WLST require at least two concordant predictors [4,27]. Accordingly, the 72–96 h time window for MRI used in our study is consistent with that used in clinical practice and is preferable to earlier imaging, as MRI beyond 7 days after ROSC may be confounded by pseudo-normalization [28,29].

MRI provided superior prognostic information compared to CT. MRI offers greater soft-tissue contrast and higher resolution, particularly when using T2-weighted fat-suppressed sequences, which allow clearer delineation of the optic nerve sheath [12,30,31,32]. Contrastingly, CT is limited by thicker slices, lower tissue contrast, and susceptibility to partial volume effects, particularly in the orbital region. These technical constraints may hinder the accurate visualization of the optic nerve sheath. Indeed, none of the CT-based metrics in our study were significantly associated with neurological outcome at either time-point, despite exhibiting excellent intra-rater reproducibility. Nevertheless, when considering prognostic accuracy, technical advantages of MRI did not translate into a high level of discriminative performance. When MRI is accessible, ONSD evaluation should preferably be performed on delayed MRI during the subacute window, as CT-based ONSD showed no significant prognostic association in this cohort despite high reproducibility. However, its discriminative ability remained modest (AUC ~0.65–0.67), underscoring that ONSD should not be used as a stand-alone tool but rather integrated with established prognostic markers.

Interestingly, intra-rater reliability was generally higher for CT than MRI in this study. This likely reflects CT’s geometric stability and the presence of well-defined bony landmarks, which facilitate consistent measurements. Conversely, MRI, while superior in visualizing soft tissue, is more susceptible to motion artifacts and signal variability, particularly in critically ill patients. These results align with prior studies suggesting that CT may offer more repeatable measurements, though with reduced sensitivity to subtle pathological changes [33].

At 72 h, NSE correlated weakly with ONSD measured on delayed MRI (r = 0.24; *p* = 0.01). Given the small effect size and absence of significant correlations for ONSD/ETD and CT-derived measures, these findings offer limited support for a mechanistic link between intracranial-pressure surrogates and neuronal injury biomarkers and should be interpreted with caution. As we did not adjust for multiple comparisons, the statistical significance may partly reflect chance.

Our findings suggest that although ONSD—especially when measured on delayed MRI—may provide useful supportive information, it is insufficient as a standalone tool for neurological prognostication. The modest AUC values indicate overlap between outcome groups, limiting its clinical discriminative value. Furthermore, the prognostic performance of ONSD was not directly compared with other established prognostic markers, such as DWI/ADC, GWR, or serum biomarkers. Therefore, its relative value and additive contribution cannot be fully determined, and the results should be interpreted with this limitation in mind. In centers performing neuroprognostication after cardiac arrest, ONSD is best measured on MRI at 72–96 h post-ROSC and interpreted as a risk flag rather than a stand-alone determinant. Values above our study’s data-driven cut-offs (e.g., second-MRI ONSD > 5.15 mm or ONSD/ETD > 0.21) may warrant heightened vigilance when corroborated by H72 NSE, reduced GWR, and DWI/ADC abnormalities, whereas ultra-early ONSD (and CT-based ONSD at either time point) should not be overinterpreted for outcome prediction.

This study had several limitations. First, this was a single-center retrospective study with a relatively modest sample size, which may limit generalizability. In addition, the study population consisted of a Korean cohort treated under a specific institutional TTM protocol and imaging schedule, which may have influenced the results and limited its extrapolation to other healthcare systems. Second, not all patients underwent both early and delayed imaging—often due to early death or clinical instability—raising the possibility of selection bias. Third, all measurements were performed by a single rater, precluding assessment of inter-rater reliability. Although intra-rater reliability was high across CT and MRI, the absence of inter-rater evaluation limits the generalizability to routine clinical practice. Fourth, we conducted a complete-case analysis without imputation, which may introduce bias if data were not missing at random. Fifth, we did not adjust for multiple comparisons, which may have increased the risk of Type I error. Sixth, the biomarker correlation analyses were exploratory; the weak association between NSE and ONSD (r = 0.24) limits causal inference and should therefore be interpreted with caution. Seventh, the use of a fixed measurement point (3 mm posterior to the globe) may not account for individual anatomical variation in optic nerve sheath morphology, which could affect the absolute ONSD values and limit generalizability. Finally, the use of specific MRI sequences may limit generalizability to other imaging protocols. Despite these limitations, to our knowledge, our study is among the first to directly compare the prognostic performance of CT and MRI obtained within 6 h and again at 72–96 h after ROSC in comatose OHCA survivors. These findings may therefore serve as valuable reference data for future research in this field. Future multicenter studies with standardized imaging and multi-rater assessments are warranted to validate and extend these findings.

## 5. Conclusions

Delayed MRI–based ONSD and ONSD/ETD demonstrated statistically significant yet modest prognostic value (AUC ~0.65–0.67), indicating substantial overlap between outcome groups and limited clinical discriminative ability. Accordingly, ONSD should not be used as a stand-alone determinant for neuroprognostication or WLST decisions. Its practical value appeared to be the greatest when measured 72–96 h post-ROSC and interpreted as a supportive marker alongside DWI/ADC, GWR, and serum biomarkers. Furthermore, as ONSD was not directly compared with other established prognostic markers in this cohort, its relative prognostic contribution remains uncertain. Given the single-center design, incomplete imaging at certain time points, lack of inter-rater reliability, and protocol-specific MRI sequences, external validation—as well as comparative and multivariable studies using standardized protocols—are warranted to better define the additive role of ONSD within the multimodal prognostic algorithms.

## Figures and Tables

**Table 1:** Baseline characteristics of the study population.

Characteristic	Overall Cohort (*n* = 136)	Good Neurological Outcome (*n* = 58)	Poor Neurological Outcome (*n* = 78)	*p*-Value *
Age, median (IQR), y	59.5 (45.0–70.0)	60.5 (44.5–68.3)	58.5 (46.5–70.5)	0.61
Sex				
Female	37 (27.2)	10 (27.0)	27 (73.0)	0.02
CCI	2.5 (1.0–4.0)	2.0 (1.0–4.25)	3.0 (1.0–4.0)	0.73
Cardiac arrest characteristics				
Witnessed	80 (61.1)	48 (86.0)	32 (41.9)	<0.001
Bystander CPR	99 (70.2)	51 (82.5)	48 (60.8)	<0.001
Shockable rhythm	47 (34.6)	36 (62.1)	11 (14.1)	<0.001
Cardiac etiology	53 (39.0)	36 (62.1)	17 (21.8)	<0.001
No flow time, median (IQR), min	2.0 (0.0–14.0)	1.0 (0.0–2.0)	11.0 (1.0–23.0)	<0.001
Low flow time, median (IQR), min	20.0 (10.0–30.0)	11.0 (8.0–18.0)	28.0 (19.8–38.3)	<0.001
rCAST score	10.5 (7.5–15.0)	6.8 (3.4–10.0)	13.8 (10.0–16.1)	<0.001
Time to examinations, median (IQR), h				
ROSC to first CT, h	1.3 (0.9–2.4)	1.1 (0.7–1.8)	1.6 (1.0–2.6)	0.03
ROSC to second CT, h	76.1 (72.3–77.9)	75.8 (74.4–77.6)	76.4 (74.0–78.0)	0.65
ROSC to first MRI, h	3.1 (2.1–4.1)	2.7 (1.9–3.9)	3.2 (2.1–4.2)	0.18
ROSC to second MRI, h	77.9 (76.3–79.7)	77.9 (75.7–79.7)	78.0 (76.5–79.7)	0.90
Neuro-prognostication				
GWR value	1.24 (1.19–1.30)	1.28 (1.22–1.31)	1.21 (1.16–1.27)	<0.001
NSE levels (H0), ng/mL	30.5 (22.3–53.5)	24.4 (19.8–30.5)	43.2 (28.7–83.5)	<0.001
NSE levels (H72), ng/mL	26.8 (16.8–120.0)	17.3 (12.6–24.1)	106.0 (32.6–262.6)	<0.001

* *p*-values are based on *χ*^2^ test for categorical variables and Mann–Whitney U test for continuous variables. IQR, interquartile range; CCI, Charson Comorbidity Index; CPR, cardiopulmonary resuscitation; ROSC, return of spontaneous circulation; rCAST, Revised Post-Cardiac Arrest Syndrome for Therapeutic Hypothermia; CT, computed tomography; MRI, magnetic resonance imaging; GWR, gray-white matter ratio; NSE, neuron-specific enolase; H0, immediately after ROSC; H72, 72 h after ROSC.

**Table 2:** CT- and MRI-based ONSD and ONSD/ETD measurements before and after TTM.

Modalities	Times	Measurement	Direction	Coefficient (95% CI)
CT	First	Optic nerve sheath diameter	Lt	0.93 (0.90–0.95)
			Rt	0.92 (0.89–0.94)
		Eyeball transverse diameter	Lt	0.75 (0.66–0.81)
			Rt	0.76 (0.68–0.82)
	Second	Optic nerve sheath diameter	Lt	0.79 (0.69–0.87)
			Rt	0.86 (0.78–0.91)
		Eyeball transverse diameter	Lt	0.82 (0.74–0.88)
			Rt	0.87 (0.81–0.92)
MRI	First	Optic nerve sheath diameter	Lt	0.80 (0.73–0.86)
			Rt	0.78 (0.71–0.84)
		Eyeball transverse diameter	Lt	0.77 (0.70–0.83)
			Rt	0.73 (0.64–0.80)
	Second	Optic nerve sheath diameter	Lt	0.73 (0.63–0.81)
			Rt	0.74 (0.63–0.81)
		Eyeball transverse diameter	Lt	0.77 (0.69–0.84)
			Rt	0.76 (0.66–0.83)

CI, confidence interval; CT, computed tomography; Lt, left; MRI, magnetic resonance imaging; Rt, right.

**Table 3:** Comparison of imaging metrics between good and poor outcome groups.

	Good Neurological Outcome (*n* = 58)	Poor Neurological Outcome (*n* = 78)	*p*-Value ^a^
First CT, 136 ^b^			
ONSD, median (IQR, mm)	4.44 (3.70–5.43)	4.51 (3.53–5.47)	0.75
ONSD/ETD, median (IQR)	0.22 (0.17–0.26)	0.21 (0.17–0.26)	0.83
Second CT, 71 ^b^			
ONSD, median (IQR, mm)	4.62 (3.98–5.01), 33 ^b^	4.57 (3.54–4.96), 38 ^b^	0.45
ONSD/ETD, median (IQR)	0.22 (0.20–0.25), 33 ^b^	0.21 (0.18–0.24), 38 ^b^	0.21
First MRI, 136 ^b^			
ONSD, median (IQR, mm)	5.31 (4.97–5.64)	5.30 (5.08–5.71)	0.68
ONSD/ETD, median (IQR)	0.23 (0.21–0.24)	0.23 (0.22–0.24)	0.53
Second MRI, 104 ^b^			
ONSD, median (IQR, mm)	5.12 (4.81–5.49), 52 ^b^	5.37 (5.17–5.74), 52 ^b^	0.003
ONSD/ETD, median (IQR)	0.22 (0.21–0.23), 52 ^b^	0.23 (0.22–0.24), 52 ^b^	0.01

^a^, *p*-values are based on Mann–Whitney U test for continuous variables. ^b^, Number of patients included in the analysis. CT, computed tomography; ONSD, optic nerve sheath diameter; ETD, Eyeball transverse diameter; IQR, interquartile range.

**Table 4:** Prognostic performance of ONSD and ONSD/ETD ratio for predicting poor outcome.

**Measurement**	**Neurological Outcome**	**First CT (*n* = 136)**	**Second CT (*n* = 71)**	***p*-Value ^a^**
ONSD, median (IQR, mm)	Good outcome	4.44 (3.70–5.43), 58 ^b^	4.62 (3.98–5.01), 33 ^b^	0.58
	Poor outcome	4.51 (3.53–5.47), 78 ^b^	4.57 (3.54–4.96), 38 ^b^	0.28
ONSD/ETD ratio, median (IQR)	Good outcome	0.22 (0.17–0.26), 58 ^b^	0.22 (0.20–0.25), 33 ^b^	0.95
	Poor outcome	0.21 (0.17–0.26), 78 ^b^	0.21 (0.18–0.24), 38 ^b^	0.52
		**First MRI (*n* = 136)**	**Second MRI (*n* = 104)**	***p*-Value ^a^**
ONSD, median (IQR, mm)	Good outcome	5.31 (4.97–5.64), 32 ^b^	5.12 (4.81–5.49), 52 ^b^	<0.001
	Poor outcome	5.30 (5.08–5.71), 44 ^b^	5.37 (5.17–5.74), 28 ^b^	0.17
ONSD/ETD ratio, median (IQR)	Good outcome	0.23 (0.21–0.24), 58 ^b^	0.22 (0.21–0.23), 52 ^b^	0.005
	Poor outcome	0.23 (0.22–0.24), 78 ^b^	0.23 (0.22–0.24), 52 ^b^	0.22

^a^, *p*-values are based on Wilcoxon signed-rank test for the comparison between the first and second imaging. ^b^, Number of patients included in the analysis. CT, computed tomography; ONSD, optic nerve sheath diameter; ETD, Eyeball transverse diameter; IQR, interquartile range.

**Table 5:** Prognostic performance of ONSD and ONSD/ETD for poor neurological outcome.

Predictor	Cut-Off	AUC (95% CI)	Sensitivity (95% CI)	Specificity (95% CI)	PPV (95% CI)	NPV (95% CI)	*p*-Value
CT							
First ONSD	≤6.74	0.52 (0.41–0.63)	98.5 (91.8–100)	4.8 (0.6–16.2)	61.9 (60.1–63.6)	66.7 (15.8–95.5)	0.74
First ONSD/ETD	≤0.4	0.51 (0.40–0.62)	100.0 (94.6–100)	0.0 (0.0–8.4)	61.1 (61.1–61.1)	No data	0.86
Second ONSD	≤4.7	0.55 (0.42–0.69)	65.8 (48.6–80.4)	48.5 (30.8–66.5)	59.5 (49.6–68.7)	55.2 (41.2–68.4)	0.44
Second ONSD/ETD	≤0.21	0.59 (0.45–0.72)	55.3 (38.3–71.4)	66.7 (48.2–82.0)	65.6 (52.1–77.0)	56.4 (45.8–66.5)	0.21
MRI							
First ONSD	>4.97	0.52 (0.42–0.62)	88.5 (79.2–94.6)	25.9 (15.3–39.0)	61.6 (57.5–65.6)	62.5 (44.0–78.0)	0.70
First ONSD/ETD	>0.19	0.52 (0.42–0.62)	98.7 (93.1–100)	1.7 (0.0–9.2)	57.5 (56.4–58.5)	50.0 (6.0–94.0)	0.69
Second ONSD	>5.15	0.67 (0.56–0.78)	82.7 (69.7–91.8)	53.9 (39.5–67.8)	64.2 (56.6–71.1)	75.7 (62.0–85.6)	0.002
Second ONSD/ETD	>0.21	0.65 (0.55–0.75)	86.5 (74.2–94.4)	42.3 (28.7–56.8)	60.0 (53.7–66.0)	75.9 (59.5–87.0)	0.005

Unit—ONSD: mm; ONSD/ETD: unitless. ONSD, optic nerve sheath diameter; ETD, Eyeball transverse diameter; AUC, area under curve; CI, confidence interval; PPV, positive predictive value; NPV, negative predictive value; CT, computed tomography; MRI, magnetic resonance imaging.

## Data Availability

The data presented here are available on request from the corresponding author. The data are not publicly available due to ethical concerns.

## Abbreviations

The following abbreviations are used in this manuscript:

ADC	Apparent diffusion coefficient
AUC	Area under the receiver operating characteristic curve
CCI	Charlson Comorbidity Index
CI	Confidence interval
CNUH	Chungnam National University Hospital
CPC	Cerebral performance category
CPR	Cardiopulmonary resuscitation
CT	Computed tomography
DWI	Diffusion-weighted imaging
ECMO	Extracorporeal membrane oxygenation
ETD	Eyeball transverse diameter
GWR	Gray-to-white matter ratio
H0	Immediately after return of spontaneous circulation
H72	72 h after return of spontaneous circulation
HIBI	Hypoxic–ischemic brain injury
ICC	Intraclass correlation coefficients
ICP	Intracranial pressure
IQR	Interquartile range
MRI	Magnetic resonance imaging
NPV	Negative predictive value
NSE	Neuron-specific enolase
ONSD	Optic nerve sheath diameter
OHCA	Out-of-hospital cardiac arrest
PPV	Positive predictive value
rCAST	Revised Cardiac Arrest Syndrome for Therapeutic hypothermia
ROC	Receiver operating characteristic
ROSC	Return of spontaneous circulation
TTM	Targeted temperature management
WLST	Withdrawal of life-sustaining therapy
